# The Effect of Kinematic Conditions and Synovial Fluid Composition on the Frictional Behaviour of Materials for Artificial Joints

**DOI:** 10.3390/ma11050767

**Published:** 2018-05-10

**Authors:** David Nečas, Martin Vrbka, Ivan Křupka, Martin Hartl

**Affiliations:** Faculty of Mechanical Engineering, Institute of Machine and Industrial Design, Brno University of Technology, Technická 2896/2, 616 69 Brno, Czech Republic; martin.vrbka@vut.cz (M.V.); krupka@fme.vutbr.cz (I.K.); martin.hartl@vut.cz (M.H.)

**Keywords:** joint replacement, friction, material, proteins, kinematic conditions

## Abstract

The paper introduces an experimental investigation of frictional behaviour of materials used for joint replacements. The measurements were performed using a ball-on-disc tribometer, while four material combinations were tested; metal-on-metal, ceramic-on-ceramic, metal-on-polyethylene, and ceramic-on-polyethylene, respectively. The contact was lubricated by pure saline and various protein solutions. The experiments were realized at two mean speeds equal to 5.7 mm/s and 22 mm/s and two slide-to-roll ratios, −150% and 150%. It was found that the implant material is the fundamental parameter affecting friction. In general, the metal pair exhibited approximately two times higher friction compared to the ceramic. In particular, the friction in the case of the metal varied between 0.3 and 0.6 while the ceramic pair exhibited friction within the range from 0.15 to 0.3 at the end of the test. The lowest friction was observed for polyethylene while it decreased to 0.05 under some conditions. It can be also concluded that adding proteins to the lubricant has a positive impact on friction in the case of hard-on-hard pairs. For hard-on-soft pairs, no substantial influence of proteins was observed. The effect of kinematic conditions was found to be negligible in most cases.

## 1. Introduction

Total joint arthroplasty is an efficient and well-established surgical procedure improving the life of patients suffering from joint diseases. According to the Health at a Glance 2015 report [1], 161 operations per 100,000 inhabitants were performed in Organisation for Economic Co-operation and Development (OECD) countries in 2013. However, despite the rapid improvement of the implanted materials in the last few decades [2], limited longevity is still recognized as the main drawback of the replacements. It should be highlighted that failure of the implant is accompanied by the need for a revising operation, which is an economic burden and substantially deteriorates the comfort of the patient. As the number of young active people with artificial joints still increases [3], it is necessary to ensure a sufficient service-life of implants to avoid any inconvenience associated with its improper function. Since the main cause of the failure is osteolysis [4] followed by aseptic loosening, the tribological performance of implants has to be understood in the context of the further development of replacements.

Several aspects have to be taken into account considering tribological processes within artificial joint contact. Mavraki and Cann [5] employed a ball-on-disc configuration when evaluating the coefficient of friction (CoF) as a function of mean speed. Metal-on-metal contact was investigated, while several test lubricants were tested. In general, CoF was kept between 0.1 and 0.4 while the test fluid had a significant impact on results. It was found that bovine serum (BS) and simple protein solutions lead to a reduction of friction in a slow-speed regime. It was also highlighted that the choice of buffer solution affects the lubrication conditions. Finally, it was concluded that friction as well as lubricating film thickness are time and sliding-distance dependent, indicating the importance of the adsorbed protein layer on the surfaces.

One of the first studies which was focused on the influence of lubricant composition on friction between polymer and metal surfac was undertaken by Yao et al. [6]. A pin-on-disk tribometer was used for the evaluation of CoF, while various test fluids were used as the lubricants; particularly deionized water, diluted (25%, 50%, 75%) and undiluted bovine calf serum (BCS), bovine synovial fluid (SF) and human periprosthetic SF. A significant variance of CoF results was observed, attributed mainly to the substantial changes of protein concentrations and rheological properties of the fluids. The lowest friction (≈0.013) was exhibited by human SF followed by deionized water. Remarkably, higher friction was detected for bovine SF and undiluted BCS (≈0.024). The highest CoF was detected for 25% BCS, which was around 0.028. Scholes and Unsworth [7] demonstrated the importance of lubricant composition in relation to the material combination of the implants. The authors tested various model fluids finding that the highest friction can be detected in the case of metal-metal implants. No significant difference between the ceramic-ceramic and metal-polyethylene pair was observed. Although there was a rapid influence of the model fluid on the CoF, the effect of proteins could not be generalized, since both decrease and increase of friction could be observed dependent on implant material. The role of individual constituents contained in SF was demonstrated by Sawae et al. [8]. The contact between an ultra-high molecular weight polyethylene (UHMWPE) pin sliding against metal/ceramic disc was studied, while a clear positive effect of hyaluronic acid added to saline solution on friction was detected.

Moreover, it was well reported that the frictional behaviour of the contact pair is substantially influenced by the surface conditions. Widmer et al. [9], investigated the ceramic-polyethylene sliding pair, finding that the oxygen-plasma based treatment of polyethylene (PE) led to improved abilities of protein adsorption onto rubbing surfaces, thus reducing both static and dynamic friction. Subsequently, it was pointed out that conformational changes have to be taken into account [10]. The authors showed that hydrophilic nature of the surfaces supports adsorption of the proteins in a native state, forming thicker lubricating film leading to lower friction between the components. An extensive investigation of the effect of model fluid composition on CoF dependent on kinematic conditions was provided in our previous study [11]. Reciprocating pin-on-plate (metal-UHMWPE) test was conducted, showing that the kinematic conditions have a crucial impact on the conformational changes of proteins, thus influencing frictional behaviour of the contact pair. At a slow speed regime (10 mm/s), a very slight increase of friction with increasing protein concentration was observed for both albumin and γ-globulin solution. The highest friction occurred when a mixture of the mentioned proteins was used as the test lubricant. An increase of sliding speed to 50 mm/s caused just a negligible change of friction. Independent of sliding speed, CoF was substantially higher when comparing protein solutions (≈0.17–0.27) with phosphate-buffered saline (PBS) without any constituents. In that case, friction was lower than 0.05. These results are in good agreement with the observation provided previously [12,13]. In the study mentioned, the effect of albumin undergoing conformational changes on the friction between the UHMWPE pin and stainless steel plate was evaluated. A substantial effect of the applied load on the results was observed; while at low loads, post-friction degraded albumin added to PBS led to a decrease of friction compared to fresh protein. The opposite behaviour occurred at higher loads. Based on the above references [11,12], it can be concluded that friction between the rubbing surfaces is affected by both kinematic and loading conditions.

The effect of replacement geometry has to be taken into account as well. Dowson et al. [14] conducted a predictive numerical analysis of friction and wear for metal-metal implant, finding that the frictional torque increases with increasing nominal diameter; however, it is affected just a little by the initial clearance between the surfaces. An experimental study of the effect of diametric clearance together with the role of model fluid was undertaken by Brockett et al. [15]. Nominal diameter of the implant was 54.6 mm, while three different clearances were considered; 53 μm, 94 μm, and 194 μm, respectively. The lowest friction factor was detected for the medium size of the clearance. Lowering the clearance led to a negligible increase of friction; however, in the case of the highest clearance, a substantial increase of friction to almost double was detected. Regarding the model fluid, 25% and 100% BS were tested, with the finding that lower friction could be reached in the case of higher protein concentration, in general, which is in a good agreement with one of the previous studies [6]. Finally, an experimental investigation of the friction coefficient using a pendulum hip joint simulator focusing on the effect of implant material and diameter was conducted by Vrbka et al. [16]. This applied 25% BS of a total protein concentration of 22.4 mg/mL, testing 28 mm and 36 mm metal-UHMWPE, ceramic-UHMWPE and ceramic-ceramic pairs. The highest CoF (≈0.16) was detected in the case of the metal-UHMWPE pair with no clear effect of nominal diameter. Ceramic-UHMWPE exhibited lower friction (≈0.14), while a larger diameter led to a further decrease (≈0.13). The lowest friction (0.12) was detected in the ceramic-ceramic pair with the same effect of diameter as in the previous case.

Based on the above references, it is apparent that the tribological behaviour of artificial joints is influenced by several aspects, such as composition of the model SF, implant material, geometry, kinematic and loading conditions. However, most of the authors focused on one particular material combination or specific composition of the test lubricant. Therefore, the aim of the present paper is to provide a detailed analysis of frictional behaviour considering various implant materials, kinematic conditions, and the composition of model synovial fluid. According to the authors’ best knowledge, such an extensive investigation has not been performed before.

## 2. Materials and Methods

**Figure 1:**
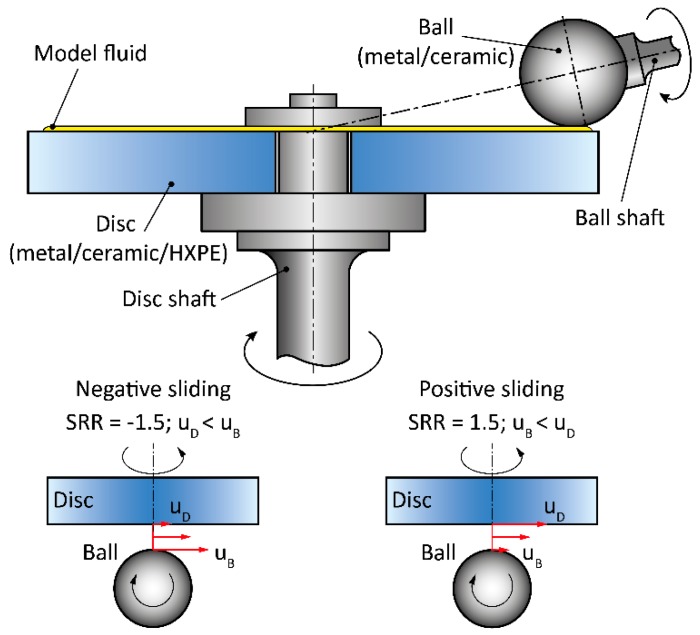
Schematic illustration of the measurement configuration.

The measurements of the CoF were conducted utilizing a commercial ball-on-disc test device Mini Traction Machine (MTM, PCS Instruments, London, United Kingdom). Both the components, the ball and the disc, could be driven independently, thus enabling the application of various kinematic conditions. The mean dynamic (sometimes also called kinetic or sliding) friction coefficient [17] is evaluated based on the ratio of friction and normal force measured by the load cell. CoF was evaluated with the acquisition frequency of 0.1 Hz. A contact pair consisted of metal (cobalt-chromium-molybdenum—CoCrMo) and alumina ceramic balls of diameter 19.05 mm sliding against 46 mm-diameter discs of metal, ceramic, and highly-crosslinked PE (HXPE), respectively. The thickness of the discs was 6 mm. A schematic illustration of the measurement configuration is displayed in Figure 1. Initial surface topography of the test specimens was checked using the phase-shifting interferometry method (Contour GT-X8, Bruker, Billerica, MA, USA). Five points which were expected to be in the contact drag of each sample were measured, while the surface roughness results are shown in Table 1.

To clarify the effect of kinematic conditions, the experiments were realized under two different mean speeds, 5.7 mm/s and 22 mm/s. Mean speed represents the average value of the ball and the disc speed under various rolling/sliding conditions. In the following text, the terms positive and negative sliding are used. In the case of negative sliding, the ball rotates faster than the disc. By contrast, under positive sliding the disc is faster. The course of the speeds of the individual components considering negative and positive sliding is demonstrated using the red arrows in the bottom part of Figure 1. Regarding the human body, it can be assumed that the hip joint operates under pure negative sliding conditions, which means that the cup is stationary, while the head rotates. Considering the ball-on-disc configuration, pure sliding represents bit severe conditions and so significant wear of the samples apparently influencing the results of CoF could be expected. Therefore, the experiments were realized under partial sliding of 150%. To avoid inaccuracies coming from the slippage of the components, the tests were realized under both positive (150%) and negative (−150%) sliding. The slide-to-roll ratio (SRR) which determines the level of slip, can be easily determined following the equation SRR = 2(u_D_ − u_B_)/(u_D_ + u_B_), where u_d_ is the speed of the disc and u_b_ is the speed of the ball. Combined rolling/sliding conditions normally occur in the knee joint; however, it should be highlighted that the present study is not focused on one particular joint type, but on the general assessment of frictional behaviour of materials used for joint implants. Applied load was 0.5 N in the case of the metal and 0.4 in the case of the ceramic balls, leading to the following contact pressures: metal-on-metal (264.7 MPa), metal-HXPE (9.9 MPa), ceramic-ceramic (280.5 MPa), ceramic-HXPE (9.5 MPa).

The main interest of the current study was in the effect of the composition of model fluid. Therefore, five test lubricants were prepared while the results were compared with those for pure PBS. The model fluids contained the dominant proteins of SF; albumin and γ-globulin, respectively. Lubricants were prepared 24 h prior to the tests and were stored in a refrigerator to enable a complete solution of the proteins in PBS. Bovine serum albumin (9048-46-8, Biomol GmbH, Hamburg, Germany) and γ-globulin from human blood (G4386, Sigma Aldrich, Darmstadt, Germany) were added to PBS in various concentrations, while the simple protein solutions were investigated as well. A summary of the test fluids is provided in Table 2. Despite the ability of the test device to control the temperature of the pot, the experiments were realized under ambient laboratory temperature of 22 °C, since it was previously referenced in literature that the increase to body temperature does not influence the results substantially [18]. The experiments were conducted under fully flooded conditions to avoid any effects coming from starvation of the contact. Eighty experiments were realized in total, while Table 3 provides the complete overview of the performed tests for better orientation. The detailed information about the speeds of the ball and the disc during various tests is given in Table 4. Random tests were conducted three times to check the repeatability of the results. Since there was excellent data agreement in terms of both the trends and the absolute values of CoF (standard deviation not larger than 0.04), the rest of the experiments were performed only once.

## 3. Results and Discussion

### 3.1. The Effect of Material and Kinematic Conditions

**Figure 2:**
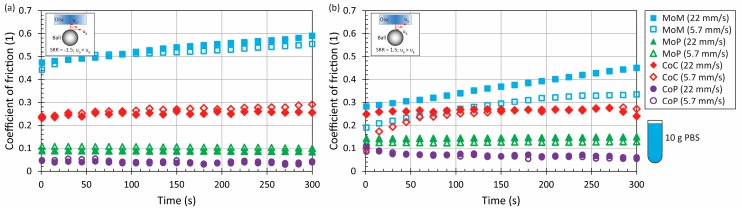
Development of coefficient of friction (CoF) as a function of time for four material combinations at lower and higher mean speed under negative (**a**) and positive (**b**) sliding conditions. Contact is lubricated by phosphate-buffered saline (PBS).

Initially, the experiments were performed with pure PBS for all the material combinations, considering both positive and negative sliding at lower (5.7 mm/s) and higher (22 mm/s) speed. The results showed that independent of kinematic conditions, the MoM combination exhibits the highest friction; however, this effect was significant mainly under negative sliding, when the friction at the end of the experiment reached almost 0.6. By contrast, the lowest friction was detected for MoP and CoP, while in the case of CoP, the friction level was less than 0.1, particularly around 0.05 for the dominant period of the experiments, which is in a good correlation with previous observations [11,19,20]. CoC frictional behaviour was found to be somewhere between, while the friction reached about 0.25. Nevertheless, it should be taken into account that the surface roughness of the employed ceramic disc was an order of magnitude higher than the surface of the ball, which could affect the results. Based on the previous findings, it might be expected that if realistic roughness of the disc could be achieved, the friction coefficient would be lower. However, without the experimental results, the potential level of CoF reduction can be only estimated. Nevertheless, it should be emphasized that the present study is rather comparative, not predicting the friction within real implants. With the exception of MoM under positive sliding conditions, there was not a substantial effect of speed. Considering the effect of time, only in the case of MoM was the development of friction with time observed. For the rest of the material combinations, CoF was stable without any fluctuations over the entire experiment. All the results can be seen in Figure 2. It should be noted that even the acquisition frequency was 0.1 Hz; for better clarity of the results, the plotted dots represent average CoF in particular time steps (1 s, 15 s, 30 s … 300 s). The inset figures are shown to illustrate the kinematic conditions. The red arrows represent the speed course. 

**Figure 3:**
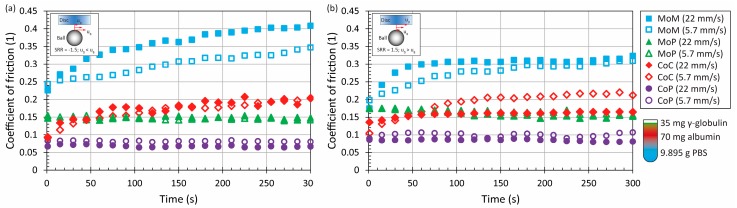
Development of CoF as a function of time for four material combinations at lower and higher mean speed under negative (**a**) and positive (**b**) sliding conditions. Contact is lubricated by model fluid Alb70+Glob35.

**Figure 4:**
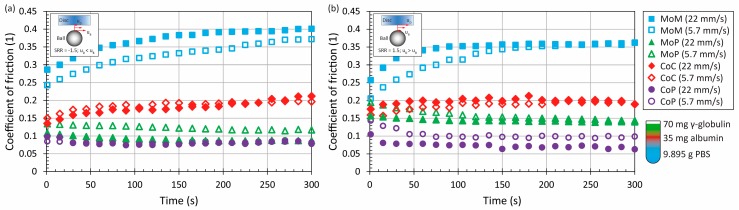
Development of CoF as a function of time for four material combinations at lower and higher mean speed under negative (**a**) and positive (**b**) sliding conditions. Contact is lubricated by model fluid Alb35+Glob70.

Adding the proteins into the saline solution in relatively low concentration (10.5 mg/mL in total) led to a slight change of CoF evolution, see Figure 3 and Figure 4 Compared to the pure PBS results, time-dependent behaviour was observed for MoM and also for CoC contact pairs, while the maximum at the end of the test was from 0.3 to 0.4 for metal. For ceramic, the friction level was about 0.2, while the very same behaviour was observed in literature, where the contact of a ceramic ball sliding against a ceramic plate lubricated by BS was analyzed [21]. To determine the influence of fluid composition, it can be seen that even if the concentration of albumin and γ-globulin was switched, see Figure 3 vs. Figure 4, almost the same results could be obtained for most of the materials and kinematic conditions. Only in the case of MoP did higher content of γ-globulin lead to a decrease of friction, as can be seen in Figure 4a.

**Figure 5:**
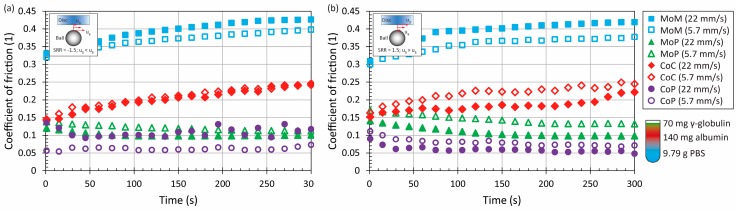
Development of CoF as a function of time for four material combinations at lower and higher mean speed under negative (**a**) and positive (**b**) sliding conditions. Contact is lubricated by model fluid Alb140+Glob70.

An increase of protein concentration to 21 mg/mL led to a very slight increase of friction for ceramic and metal pairs. In the case of MoP and CoP, the friction remained the same or decreased a little bit. However, since the change is negligible, see Figure 3 and Figure 4 vs. Figure 5, it can be concluded that the protein content does not play an important role regarding these so-called hard-on-soft bearing pairs. Finally, focusing on the particular material combinations, it can be seen that the behaviour corresponds well to the pure PBS results, while the highest friction is exhibited by MoM, followed by CoC, MoP and CoP (Figure 2, Figure 3, Figure 4 and Figure 5). The highest friction in the case of metal-metal contact was reported also by Scholes and Unsworth [7]. Nevertheless, it should be noted that the results do not completely correspond to observations provided by Vrbka et al. [16], who determined the friction coefficient for various implant pairs. In the reference, the authors measured the lowest friction when considering a ceramic head sliding against a ceramic cup. However, it must be noted that different experimental conditions were employed; in particular, BS was employed as the test lubricant, and the experiments were realized in different geometrical configurations. Moreover, as mentioned above, the roughness of ceramic discs used in the present study was an order of magnitude higher than is the roughness of real acetabular cups, which can also play a significant role.

### 3.2. The Effect of Model Synovial Fluid Composition

**Figure 6:**
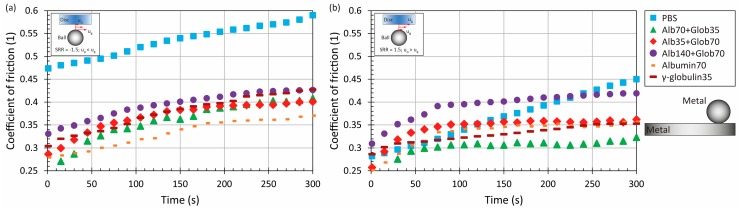
Development of CoF as a function of time for metal-on-metal pair at higher mean speed under negative (**a**) and positive (**b**) sliding conditions for various model fluids.

**Figure 7:**
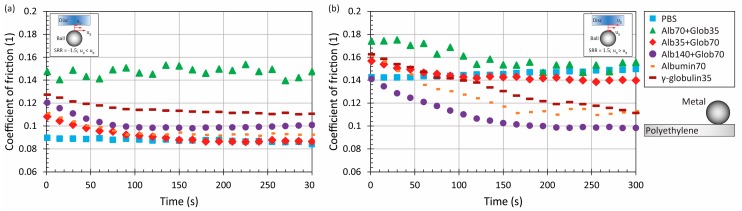
Development of CoF as a function of time for metal-on-polyethylene pair at higher mean speed under negative (**a**) and positive (**b**) sliding conditions for various model fluids.

**Figure 8:**
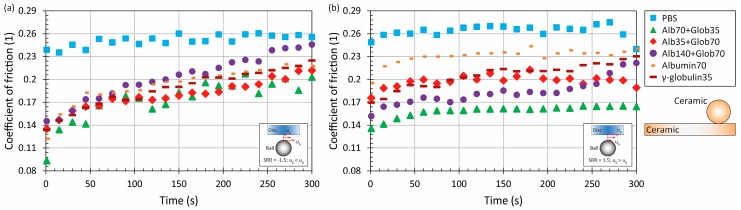
Development of CoF as a function of time for ceramic-on-ceramic pair at higher mean speed under negative (**a**) and positive (**b**) sliding conditions for various model fluids.

**Figure 9:**
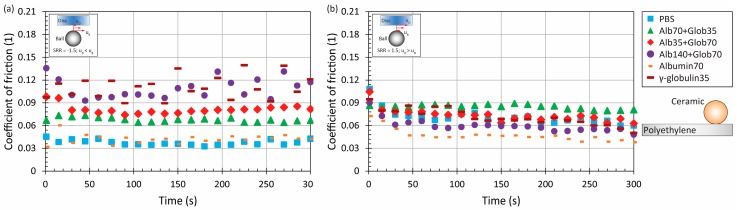
Development of CoF as a function of time for ceramic-on-polyethylene pair at higher mean speed under negative (**a**) and positive (**b**) sliding conditions for various model fluids.

To clearly identify the effect of model SF composition on frictional behaviour, the additional tests were performed at higher speed considering the simple protein solutions of albumin and γ-globulin as well. The results for individual material combinations are displayed in Figure 6, Figure 7, Figure 8 and Figure 9, respectively. For MoM contact, it can be seen that the results for pure PBS are influenced by the positivity/negativity of sliding. Under negative sliding, the level of friction is considerably higher (around 0.6 at maximum) for PBS compared to protein solution (around 0.4). In terms of individual fluids, albumin-containing lubricant exhibited the lowest friction, approximately 0.37. For the rest of the lubricants, there is no clear impact on lubrication, while the friction is kept between 0.4 and 0.43 at the end of the test, see Figure 6a. Considering the positive sliding conditions, the CoF development is slightly different. The highest friction over the dominant part of the experiment was exhibited by the solution with relatively high protein content. However, after 75 s, the friction was stabilized at about 0.4 without any further time-dependent change. The course of friction for saline solution is linearly increasing (the same behaviour as under negative sliding), reaching 0.45 at the end. For the other protein solutions, the behaviour is almost the same, with the exception of lubricant containing 70 mg/mL of albumin and 35 mg/mL of γ-globulin, which exhibits the lowest friction. This can be attributed to the action of the protein film. It was observed in literature that, considering the same model fluid as in the case of the present study, a metal head formed much thicker protein film under positive sliding, which can lead to a decrease of friction between the surfaces [22].

It is apparent that the MoM combination exhibits an increasing tendency of friction, in general. By contrast, when the disc is of polyethylene, friction is decreasing or constant, as is shown in Figure 7. In that case, the friction level is associated with the kinematic conditions. When the ball rotates faster than the disc, the lowest friction was exhibited by PBS, and the lubricant with the higher content of γ-globulin. The highest friction was observed for lubricant with lower protein concentration (10.5 mg/mL) and the dominant presence of albumin. The tendency was also quite unstable in that case. Nevertheless, it has to be taken into account that the difference between minimum and maximum friction is just 0.05; therefore, it might be assumed that the effect of the lubricant is negligible. When the disc was faster, the lowest friction was reached, once the lubricant with higher protein content (21 mg/mL) was employed. In addition, both the simple solution exhibited relatively low friction of around 0.11. What is in correlation with previous findings is the friction level for saline solution. In a recent study [23], a polyethylene disc conducted reciprocating motion against a metal pin (pure positive sliding), while the friction was from 0.12 to 0.16, which corresponds to the present results, see Figure 7b.

Considering the ceramic-ceramic contact, the friction is increasing under negative sliding as in the case of the metal-metal pair. Moreover, there is a good correlation of the effect of proteins, while adding the proteins to the saline solution led to a decrease of friction, especially during the first part of the test, see Figure 8a. The same behaviour can be observed even for positive sliding, while the effect of the lubricant is more substantial; anyway, the variance in friction is still limited. In this particular case, the friction is relatively stable for all the test fluids, as is displayed in Figure 8b. Similar friction with film thickness development can be found, as mentioned for the metal sliding pair. Using the fluorescent microscopy technique, we evaluated the central film thickness in ceramic-on-glass contact, while it was found that the lubricant layer for alumina ceramic under positive sliding was much thicker compared to negative sliding [24].

Finally, the experiments were realized with a ceramic ball and polyethylene disc. The effect of the lubricant was again very limited, as for the MoP pair. In particular under positive sliding, the role of fluid is unimportant, since the difference between minimum and maximum friction is just about 0.04, see Figure 9b. Therefore, it is not suitable to identify which lubricant leads to the lowest and the highest friction, respectively. The only noticeable finding is that in the case of negative sliding, slightly higher friction (around 0.12) was exhibited by simple γ-globulin solution and by the solution with a higher content of protein.

As could be expected based on the previous findings, the present results clearly prove that the most important factor influencing the frictional behaviour of implants is the material of the components. What is quite surprising is the limited influence of model fluid composition, mainly in the case of MoP and CoP pairs. Nevertheless, it should be taken into account that in hard-on-soft pairs significantly lower contact pressure occurs; therefore, this may be an explanation for relatively low sensitivity of friction to test fluid. Anyway, since the previous observations focusing on the assessment of film thickness in hip replacements explored whether the effect of fluid composition may be fundamental [25,26,27,28], deeper investigation of lubrication mechanisms seems to be a crucial challenge for biotribologists.

Obviously, the authors realize several limitations to the performed study. The most important point might be the geometrical arrangement. Previously, most of the tribological studies dealing with friction and film thickness evaluation were conducted considering simplified ball-on-disc (pin-on-plate) test configuration. However, our recent observations clearly indicate that the lubrication is apparently influenced by the contact conformity [29]. However, it should be noted that the main purpose of the present study was to provide a comprehensive comparison of frictional behaviour regarding implant material, the composition of model synovial fluid, and kinematic conditions. Therefore, the main findings can be generalized irrespective of the contact geometry. A similar approach was presented before in literature [5]. A later limiting point is the composition of the employed test lubricants. As discussed above, the protein content and its ratio may not be as substantial as previously expected. Although several experimental investigations focused on in vitro wear, the testing of implants could provide satisfactory wear data compared to clinical observations; it should be taken into account that our recent study [28] clearly indicates that the lubrication mechanisms are affected dominantly by the presence of hyaluronic acid and phospholipids. These findings are in a good agreement with the data published elsewhere [8,30]. The last point arising could be the effect of temperature on frictional behaviour. However, an increase of temperature of around 15 °C should not cause any change in terms of lubrication performance [18], and also conformational changes potentially influencing the results [11] are not expected. Considering the aforementioned limitations, the motivation for future study should be the necessity of applying complex model fluids containing all the synovial constituents together with attention to the real conformity of rubbing surfaces.

## 4. Conclusions

The performed study focused on the evaluation of the friction coefficient within the contact of materials used for joint replacements. The contact was lubricated by various model synovial fluids while the study was aimed at assessment of the effect of implant material and kinematic conditions in terms of speed and slide-to-roll ratio. The findings can be summarized as follows:

Based on the results obtained, it seems that the dominant factor influencing the frictional performance of joint replacements is the implant material. The effect of kinematic conditions as well as the composition of model synovial fluid was found to be much less important.

The highest friction is exhibited by metal-metal contact. For the ceramic-ceramic pair, the friction was approximately half compared to the metal pair. This behaviour was observed independent of the applied test fluid.

If the polyethylene disc was used as the counterpart, the friction was very low, less than 0.05 in some cases. Generally, the lowest friction was detected for the ceramic-polyethylene pair.

Considering the trend of friction for various fluids regarding individual material combinations, it was found that the friction has an increasing tendency in the case of hard-on-hard pairs (MoM, CoC); however, the tendency is decreasing or constant for hard-on-soft implants (MoP, CoP).

Proteins added to saline solution lead to a decrease of friction for the metal-metal pair, while for the other combinations there are no significant differences when comparing PBS results with protein solutions.

In the case of a hard-on-soft bearing pair in particular, the role of fluid compositions was found to be negligible, as the differences between minimum and maximum values at the end of the experiments are less than 0.08.

## Figures and Tables

**Table 1:** Surface topography parameters of the test samples.

Parameter	Ball		Disc		
-	Metal	Ceramic	Metal	Ceramic	HXPE
Average surface roughness Ra (μm)	0.087	0.035	0.008	0.998	0.841
Standard deviation SD (μm)	±0.00587	±0.00414	±0.00039	±0.10707	±0.13631

**Table 2:** Summary of the applied model fluids.

Model Synovial Fluid (MSF)	Volume (mL)	Protein Content (mg/mL)		Total Protein Concentration (mg/mL)
-	-	Albumin	γ-globulin	-
Alb70+Glob35	10	7	3.5	10.5
Alb35+Glob70	10	3.5	7	10.5
Alb140+Glob70	10	14	7	21
Albumin70	10	7	-	7
γ-globulin35	10	-	3.5	3.5
PBS	10	-	-	-

**Table 3:** Overview of the performed experiments.

Experiment Set	Material Combination	Mean Speed (mm/s)	Slide-To-Roll Ratio (%)	Model Synovial Fluid
1	Metal-on-Metal (MoM)	5.7; 22	150; −150	Alb70+Glob35; Alb35+Glob70; Alb140+Glob70; PBS
2	Metal-on-highly crosslinked polyethylene (HXPE) (MoP)	5.7; 22	150; −150	Alb70+Glob35; Alb35+Glob70; Alb140+Glob70; PBS
3	Ceramic-on-Ceramic (CoC)	5.7; 22	150; −150	Alb70+Glob35; Alb35+Glob70; Alb140+Glob70; PBS
4	Ceramic-on-HXPE (CoP)	5.7; 22	150; −150	Alb70+Glob35; Alb35+Glob70; Alb140+Glob70; PBS
5	MoM	22	150; −150	Albumin70; γ-globulin35
6	MoP	22	150; −150	Albumin70; γ-globulin35
7	CoC	22	150; −150	Albumin70; γ-globulin35
8	CoP	22	150; −150	Albumin70; γ-globulin35

**Table 4:** A detailed overview of the kinematic conditions.

Slide-To-Roll Ratio (%)	Mean Speed (mm/s)	Ball Speed (mm/s)	Disc Speed (mm/s)
−150	5.7	9.975	1.425
−150	22	38.5	5.5
150	5.7	1.425	9.975
150	22	5.5	38.5
